# Pneumatic wound compression after hip fracture surgery did not reduce postoperative blood transfusion: A randomized controlled trial involving 292 fractures

**DOI:** 10.1080/17453670902804893

**Published:** 2009-02-01

**Authors:** Anna Apelqvist, Markus Waldén, Gert-Uno Larsson, Isam Atroshi

**Affiliations:** ^1^Department of Orthopedics, Hässleholm-Kristianstad, Hässleholm HospitalHässleholmSweden; ^2^Department of Clinical Sciences, Lund UniversityLundSweden

## Abstract

**Background and purpose** Patients with fracture of the proximal femur often undergo blood transfusion. A pneumatic compression bandage has been shown to reduce transfusion after primary hip arthroplasty for osteoarthritis. In this randomized trial, we evaluated the efficacy of this bandage following surgery for hip fracture.

**Patients and methods** 288 patients, 50 years or older with 292 fractured hips treated with hemiarthroplasty or internal fixation (except pinning), were randomized to an experimental group with pneumatic wound compression applied after surgery (n = 138) and a control group with the same dressing but no compression (n = 154). Transfusion threshold was blood hemoglobin below 100 g/L. The primary outcome measures were the number of blood units and the proportion of patients transfused after surgery.

**Results** The primary outcome measures were similar in both groups. The mean number of postoperatively transfused blood units was 1.3 in the compression group and 1.1 in the non-compression group. Blood transfusion was given to 84 patients (62%) in the compression group and to 85 patients (55%) in the non-compression group.

**Interpretation** Pneumatic wound compression does not reduce the need for transfusion after hip fracture surgery.

## Introduction

Bleeding from a hip fracture caused by the injury itself and by the subsequent surgery often necessitates blood transfusion. To reduce bleeding during and after major orthopedic surgery, such as hip and knee arthroplasty, various methods (mainly pharmaceutical) have been investigated (Keating and Meding 2002, Moonen et al. 2006). A pneumatic wound compression bandage has been introduced as a new method to reduce postoperative bleeding after hip arthroplasty (Hörnberg et al. 2002). It was evaluated and found to reduce the amount of blood transfusion in two small randomized studies on primary total hip arthroplasty (THA) for hip osteoarthritis (OA) (Hörnberg et al. 2002, Johansson et al. 2005a). We have not found any published studies that used this pneumatic compression bandage in patients undergoing surgery for hip fracture. The primary objective of this randomized controlled trial was therefore to assess the efficacy of the pneumatic compression bandage, applied immediately after hip fracture surgery, in reducing the need for blood transfusion.

## Patients and methods

Patients were recruited at the Emergency Department of Kristianstad Hospital, Sweden, from January 2005 through December 2006. The department provides emergency care for a region with a population of 170,000. Patients with a femoral neck fracture, or pertrochanteric or subtrochanteric fracture were screened by an orthopedic surgeon for enrollment in the trial. Those who fulfilled the eligibility criteria were asked to participate. Written informed consent was obtained from all participating patients. For patients who could not provide consent themselves because of cognitive impairment, the orthopedic surgeon or the ward nurse sought consent from a family member.

### Eligibility criteria

The inclusion criteria were patients 50 years or older presenting with: (1) displaced cervical femoral fracture planned for surgery with hemiarthroplasty, (2) stable pertrochanteric fracture planned for internal fixation with plate and sliding screw or twin hook, or (3) unstable pertrochanteric or subtrochanteric fracture planned for internal fixation with proximal intramedullary nail. Intracapsular femoral neck fractures treated with pinning seldom require transfusion, and they were therefore not included in our study.

**Figure:**
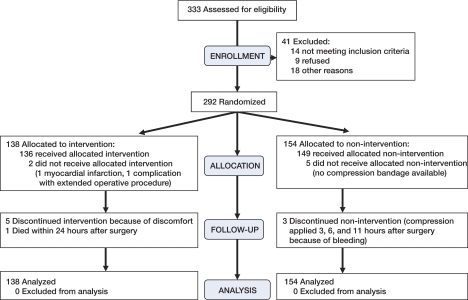


The exclusion criteria were: (1) undisplaced cervical fracture, (2) displaced cervical fracture planned for fixation with hook pins (usually in patients below 75 years of age), (3) pathologic fracture due to malignancy, (4) concomitant fractures or injuries that might require blood transfusion, and (5) refusal to undergo blood transfusion (such as Jehovah’s Witnesses).

During the study period, 745 consecutive patients with proximal femoral fracture were registered at the Emergency Department. Of 333 patients assessed for eligibility, 288 patients with 292 hip fractures met the inclusion criteria and agreed to participate in the study; 138 were randomized to the compression group and 154 to the non-compression group.

### Surgical procedures

All patients received an intravenous injection of tranexamic acid (100 mg/kg body weight) 20 min preoperatively, and a second injection after 4 h. All patients were operated on with general or spinal anesthesia.

For hemiarthroplasty, the Lubinus SP II Variokopf (Link, Hamburg, Germany) or the Exeter UHR (Stryker Howmedica Osteonics, Exeter, UK) was used. For internal fixation of stable pertrochanteric fractures, the Hansson twin-hook hip fracture system (Swemac Orthopaedics AB, Linköping, Sweden) or the Ambi/Classic compression hip screw system (Smith and Nephew, Memphis, TN) was used. For unstable pertrochanteric and subtrochanteric fractures, the Gamma intramedullary nail system (Stryker Orthopedics, Mahwa, NJ) was used.

The patient was operated upon on a fracture table with longitudinal traction (internal fixation) or in a lateral decubitus position with a dorsolateral approach without trochanter osteotomy (hemiarthroplasty). No drain was used. A single dose of cloxacillin (2 g intravenously) was given shortly before operation (600 mg clindamycin in case of allergy to penicillin). After wound closure, a small dressing was placed over the wound.

### Randomization (flow chart)

A randomization list was created based on a computer-generated random number list. Based on the list, consecutively numbered sealed, opaque envelopes were prepared. Patients were randomized into 2 groups: an intervention group wearing the bandage with pneumatic pressure applied and a control group wearing the bandage but without pressure. Randomization of group assignment was done immediately after surgery by opening the lowest-numbered envelope.

### Sample size

A previous study of 50 patients who had undergone THA for OA showed a statistically significant difference of 23% fewer transfused patients and 65% less transfused blood units in the compression group compared with the non-compression group (mean 0.6 units and 1.8 units, respectively, no SDs reported) (Hörnberg et al. 2002). In a similar study, the mean number of transfused units was 1.4 (SD 1.5) in the compression group and 2 (SD 2) in the non-compression group (Johansson et al. 2005a). Assuming an SD of 1.5, randomization of 280 patients would provide 80% power at a 5% 2-sided significance level to detect differences of at least 0.5 transfused blood units between the experimental and control groups. This sample size would also allow detection of a difference of at least 20% in the proportion of transfused patients (assuming a 70% transfusion rate in the control group).

**Figure:**
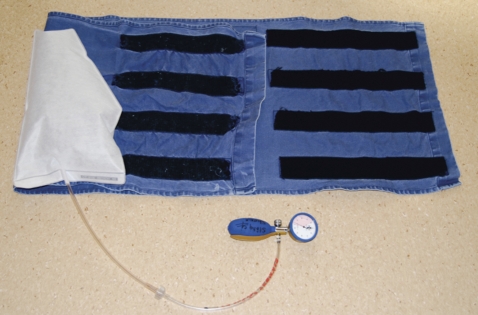
The compression bandage.

### Bandage

The pneumatic compression bandage, Calmed compression dressing system (Calmar Medical AB, Askim, Sweden), was applied when the patient was being moved from the operation table to a bed. The bandage consists of a garment placed around the hip region and an inflatable bag placed directly over the postoperative dressing on the surgical incision (Figure). In patients randomized to the compression group, air was pumped into the bag. The initial pressure of 40 mm Hg was maintained for 30 min, after which the pressure was lowered to 20 mm Hg and kept for 12 h. In patients randomized to the control group, the bandage was applied in an identical manner but the bag was not inflated.

### Postoperative care

The postoperative care followed routine clinical care. A daily subcutaneous injection of 40 mg enoxaparin was given for 10 days. Early mobilization with weight bearing as tolerated was allowed from the first postoperative day.

### Transfusion

A transfusion of 1 red cell unit was given when blood hemoglobin (Hb) was below 100 g/L, and repeated when necessary using the same threshold.

### Assessments

At baseline, data concerning preoperative use of medication with antithrombotic effect, including warfarin, acetylsalicylic acid (ASA), or other anti-platelet drugs or non-steroidal anti-inflammatory drugs (NSAIDs), were recorded. Time from arrival at the Emergency Department to surgery was recorded. Blood Hb, platelet count, international normalized ratio (INR), and activated partial thromboplastin time (APT-t) were measured at admission. Blood Hb was routinely measured 3 h after surgery and on postoperative days 1 and 5. Further Hb measurements were made on clinical suspicion of low Hb, if the Hb value was close to the transfusion threshold, or if blood had been transfused. Intraoperative blood loss was estimated by the nurse anesthetist by counting wet swabs and drapes together with suction volume minus volume of any irrigation fluid used. The number of red cell units transfused before, during, and after surgery (up to discharge from hospital or death) was recorded. Potential complications related to the bandage—including pressure ulcers, deep vein thrombosis and pulmonary embolism, and wound complications (delayed healing, necrosis, rupture or infection)—were recorded up to 3 months after surgery. The dressing was inspected daily by ward nurse assistants and the surgical wound was routinely inspected on day 5, on the day of discharge, and when the sutures were removed 2 weeks postoperatively. Data for all treated infections or positive cultures up to three months postoperatively were recorded by a nurse in a centralized hospital database. Pre-existing or new pressure ulcers were looked for and documented daily by ward nurse assistants on a special form. Clinically suspected deep vein thrombosis was examined with venography or ultrasound, and pulmonary embolism with spiral computerized tomography of the chest.

**Figure:**
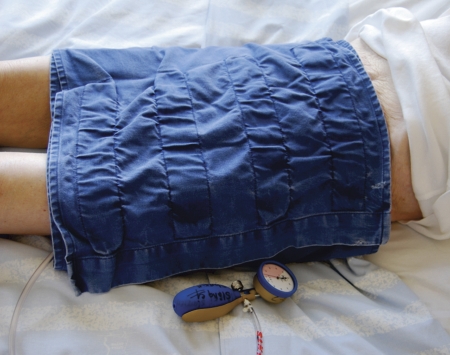
The compression bandage.

### Outcome measures

The primary outcome measure was the mean number of red cell units transfused postoperatively and the proportion of patients in each group requiring postoperative blood transfusion. The secondary outcome measure was the rate of postoperative complications.

### Ethics

The study was approved by the Medical Research Ethics Committee of Lund University (704/2004-11-30).

### Statistics

Intention-to-treat analyses were performed. The mean difference and 95% CI in number of postoperatively transfused blood units was compared between the 2 groups using the independent-samples t-test. Difference between the 2 groups in the proportion of postoperatively transfused patients was analyzed using a logistic regression. Analyses adjusting for age, sex, use of drugs with antithrombotic effect and NSAIDs, baseline hemoglobin, type of surgery, number of blood units transfused before and/or during surgery, and amount of intraoperative bleeding gave similar results, and therefore the unadjusted results have been used. Similar analyses were done after stratifying the patients according to type of surgery. Because 4 patients had been enrolled twice, as they had a subsequent fracture of the contralateral hip during the study period, a mixed-effects model analysis was performed to validate the results of the t-test and regression analyses. Complications were compared. The 30-day and 90-day mortality rates were compared between the groups. All analyses were two-sided and a p-value of < 0.05 was considered to indicate statistical significance.

## Results

### Patients

Most of the 288 patients (with 292 fractures) received the allocated intervention, but 3 patients assigned to non-compression (1 hemiarthroplasty and 2 intramedullary nail) received compression ordered by the anesthesiologist because of “excessive bleeding” and 5 patients assigned to compression (1 hemiarthroplasty, 4 sliding plate) removed the bandage because of discomfort or confusion after 1, 7, 8, and 10 hours (the time was not recorded for 1 patient). There were no differences between the 2 groups regarding their baseline characteristics (Table 1).

**Table 1.** Baseline characteristics

	Compression (n = 138)	No Compression (n = 154)
Age	83 (9)	82 (8)
Women, n (%)	100 (72)	113 (73)
Type of fracture, n (%)		
Cervical	55 (40)	68 (44)
Pertrochanteric	63 (46)	75 (49)
Subtrochanteric	20 (14)	11 (7)
Type of surgery, n (%)		
Hemiarthroplasty	45 (33) **^a^**	60 (39)
Sliding plate	42 (30)	50 (32)
Intramedullary nail	51 (37)	44 (29)
Preoperative medication, n (%)		
Acetylsalicylic acid	65 (47)	64 (42)
NSAID	8 (5.8)	6 (3.9)
Warfarin	11 (8.0)	13 (8.4)
Time from admission to surgery (h) **^b^**	21 (11)	21 (12)
Hemoglobin (g/L) at baseline	124 (15)	126 (15)
Patients transfused		
preoperatively, n (%)	20 (15)	21 (14)
intraoperatively, n (%)	47 (34)	45 (29)
Bleeding intraoperatively (mL)	351 (292)	325 (275)
Hemiarthroplasty	462 (186) **^a^**	418 (251)
Sliding plate	193 (142)	148 (85)
Intramedullary nail	383 (393)	398 (349)

Values are shown as mean (SD) unless otherwise specified.

**^a^** Including 1 total arthroplasty.

**^b^** No data were available for 14 patients in the compression group and for 13 patients in the non-compression group.

### Blood transfusion

No statistically significant differences were found between the 2 groups with regard to the mean number of postoperatively transfused units, or in the proportion of patients requiring blood transfusion (Table 2). The mixed-effects model analysis gave similar results, with a mean difference in number of postoperatively transfused units of 0.17 (95% CI: -0.13 0.46, p = 0.3) and an odds ratio of having postoperative transfusion of 1.69 (95% CI: 0.5–5.7, p = 0.4). No differences were found between the 2 groups when stratified according to type of surgery (Table 3).

**Table 2.** Results of the outcome variables

	Compression (n =138)	No compression (n = 154)	Mean difference or odds ratio (95% CI)	p-value
Blood units transfused postoperatively	1.3 (1.3)	1.1 (1.3)	0.17 (-0.13–0.46)	0.3
Patients transfused postoperatively, n (%)	85 (62)	84 (55)	1.34 (0.84–2.13)	0.2
Hemoglobin day 1 (g/L)	109 (10.3)	109 (11.2)	0.5 (-0.2–0.3)	0.7
Hemoglobin day 5 (g/L)	111 (9.7) **^a^**	113 (10.1) **^a^**	-2.1 (-4.4–0.3)	0.08

Data are shown as mean (SD) unless otherwise specified.

**^a^** No data were available for 7 patients in the compression group and for 6 in the non-compression group.

**Table 3.** Results of the primary outcome variables stratified according to surgical method

Blood transfusion postoperatively	Compression (n = 138)		No compression (n = 154)		Mean difference or odds ratio (95% CI)	p-value
Blood units, mean (SD)						
Hemiarthroplasty	n=45 **^a^**	0.87 (1.2)	n=60	0.83 (1.2)	0.03 (-0.5–0.5)	0.9
Sliding plate	n=42	1.10 (1.3)	n=50	0.98 (1.1)	0.1 (-0.4–0.6)	0.7
Intramedullary nail	n=51	1.82 (1.2)	n=44	1.68 (1.3)	0.1 (-0.4–0.7)	0.6
Patients, n (%)						
Hemiarthroplasty	n=45 **^a^**	20 (44)	n=60	26 (43)	1.1 (0.48–2.3)	0.9
Sliding plate	n=42	22 (52)	n=50	25 (50)	1.1 (0.48–2.5)	0.8
Intramedullary nail	n=51	43 (84)	n=44	33 (75)	1.8 (0.65–5.0)	0.3

**^a^** Including 1 total arthroplasty.

### Complications and mortality

No differences in complications or mortality were found between the 2 groups. Superficial wound infection occurred in 6 patients (4.3%) in the compression group and 8 (5.2%) in the non-compression group; all healed without treatment, or with antibiotics. No deep wound infections occurred. 1 patient in the non-compression group had a minor wound rupture that healed without surgery. Another patient in the non-compression group developed a blister near the surgical wound, recorded as a complication to the bandage. Sacral pressure ulcers were registered in 4 patients in the compression group and in 5 in the non-compression group. Deep vein thrombosis was diagnosed in 2 patients in each group and pulmonary embolism was diagnosed in 2 patients in the compression group.

The 30-day mortality was 8% in the compression group (n = 11) and 6.5% in the non-compression group (n = 10), and the 90-day mortality was 10% (n = 14) and 12% (n = 18), respectively. 7 patients died during the primary hospital stay, with the causes of death being cerebral embolism, myocardial infarction, heart failure, pulmonary edema, kidney failure, pneumonia, or septicemia. For the patients who died after discharge, the cause of death was not ascertained.

## Discussion

The compression bandage evaluated in our study has previously been reported to be effective in reducing the need for transfusion, but not the blood loss, following THA in patients with OA (Hörnberg et al. 2002, Johansson et al. 2005a). The former study compared 26 patients who received the compression bandage with 24 patients who received a standard wound dressing, and reported a significantly reduced amount of postoperative blood transfusion and a significantly reduced proportion of transfused patients in the compression group (Hörnberg et al. 2002). The later study compared 51 patients who had the compression bandage and no wound drainage with 54 patients who had a standard dressing and wound drainage, and it reported a significantly reduced amount of blood transfusion in the compression group but not a reduced proportion of transfused patients (Johansson et al. 2005a).

We could not demonstrate that the compression bandage was similarly effective after hip fracture surgery. One possible explanation is that OA patients and hip fracture patients differ in important factors such as age, body mass, general health status, and medications. It is common to advise OA patients to stop intake of drugs known to increase bleeding (such as ASA and NSAIDs) before surgery (Faunö et al. 1993, Hörnberg et al. 2002, Johansson et al. 2005b). In the present study, there were no differences between groups regarding the use of such drugs before surgery. In fracture patients, preoperative bleeding from the fracture itself can be considerable—a factor that is not influenced by the compression bandage.

The two studies that reported an advantage in using the compression bandage in THA used wound drain, either in both groups (Hörnberg et al. 2002) or only in the control group (Johansson et al. 2005a). Wound drain was, however, not used in our study and it is currently not commonly used after hip arthroplasty (Niskanen et al. 2000) or hip fracture surgery (Tjeenk et al. 2005) since neither the blood loss nor the wound infection rates are affected. The study by Hörnberg et al. (2002) was relatively small and did not adjust for other differences, possibly resulting in a type-1 error. The study by Johansson et al. (2005a) reported somewhat conflicting findings: a reduction in the mean number of transfused units but not in the number of patients transfused. In the light of our results, a larger randomized study may be warranted to confirm the efficacy of the compression bandage in patients undergoing primary THA for OA. However, in hip fracture surgery, our results from a large sample size did not show any evidence to suggest that the compression bandage may be effective. We used intention-to-treat analysis but only 3 patients randomized to the non-compression group received compression, so this cannot have influenced the results. In our study, we used the pressure levels and application time proposed by the manufacturer and used by Hörnberg et al. (2002), but somewhat shorter than the application time used by Johansson et al. (2005a). It is not known whether a higher pressure level and/or a longer application time might be more effective.

One limitation of our study is that many eligible patients were not enrolled because of difficulty in obtaining consent, or because they were not asked to participate by the surgeon at the Emergency Department. Many patients suffer from dementia and/or confusion on admission and are therefore incapable of giving informed consent, and it was not always possible to contact a family member. However, it seems very unlikely that this factor would have influenced the results.

To determine the need for blood transfusion, we used Hb below 100 g/L. This transfusion threshold has been used in some studies (Anekstein et al. 2004, Muir 1995) but other studies have used lower thresholds of 70–90 g/L (Marval and Hardman 2004). One pilot study randomized 84 patients with hip fracture to receive transfusion if Hb levels dropped below the predefined threshold of 100 g/L, or to receive transfusion only for symptoms of anemia or a Hb of less than 80 g/L, but no firm conclusions could be drawn from that study (Carson et al. 1998b). Consequently, there is a lack of evidence on which to base the transfusion threshold following hip fracture surgery. In the 2 studies with pneumatic wound compression in OA patients, Hörnberg et al. (2002) used a transfusion threshold of Hb below 100 g/L and Johansson et al. (2005a) used Hb below 90 g/L or “symptoms of anemia requiring transfusion”. Many authors have advocated lower thresholds (Carson et al 1998a, Madjdpour et al. 2006). Using a lower transfusion threshold in our study would have resulted in fewer transfused patients and a lower amount of transfusions in both groups, but it is unlikely that this would have influenced the results regarding the efficacy of the compression bandage.

Pressure applied to the surgical wound could theoretically give rise to local skin ischemia and thereby increase the risk of delayed healing, wound rupture, necrosis, or infection. In a study of 965 cervical hip fractures, wound infection occurred in 7% of patients receiving blood transfusion and in 4% of those not receiving blood transfusion (Levi and Sandberg 1998). In our study, the rate of superficial wound infection was 5%, which is in agreement with previous studies (Merrer et al. 2007), with no difference found between the compression and control groups. One other potential complication of using a compression bandage is the occurrence of pressure ulcers. However, both groups had equal amounts of sacral pressures and no patient developed ulcers on either hip. Compression around the hip region might theoretically cause venous stasis and increased risk of thromboembolic events. According to a Cochrane review, deep vein thrombosis occurs in up to 26% of patients following hip fracture surgery, despite prophylaxis with LMWH (Handoll et al. 2002). In our study, clinical thromboembolic complications occurred in 3.6% of patients in the compression group, which is a relatively low rate.
